# Do introductory courses disproportionately drive minoritized students out of STEM pathways?

**DOI:** 10.1093/pnasnexus/pgac167

**Published:** 2022-09-28

**Authors:** Neil Hatfield, Nathanial Brown, Chad M Topaz

**Affiliations:** Pennsylvania State University, University Park, PA 16802, USA; Pennsylvania State University, University Park, PA 16802, USA; Institute for the Quantitative Study of Inclusion, Diversity, and Equity, Williamstown, MA 01267, USA; Williams College, Williamstown, MA 01267, USA

**Keywords:** differential outcomes, degree, graduation, ethnicity, race, sex, STEM, introductory courses

## Abstract

Diversifying science, technology, engineering, and mathematics (STEM) requires a critical examination of institutional structures at every educational level. In higher education, performance in core introductory courses required for STEM degrees is strongly associated with degree completion. Leveraging a multi-institutional database, we examine nearly 110,000 student records from six large, public, research-intensive universities in order to assess whether these introductory courses disproportionately weed out underrepresented minority (URM) students. We find that the association between low performance in an introductory STEM class and failure to obtain a STEM degree is stronger for URM students than for other students, even after controlling for academic preparation in high school and intent to obtain a STEM degree. To facilitate interpretation of our multivariate logistic regression model, and to highlight the dire situation in higher education, we also calculate predicted probabilities of STEM degree attainment for students of various demographics. The probability of obtaining a STEM degree for a STEM-intending white male student with average academic preparation who receives grades of C or better in all introductory courses is 48%. In contrast, for an otherwise similar URM female student, the probability is merely 35%. If these students receive less than a C in even one introductory STEM course, the probabilities drop to 33% and 21%, respectively.

Significance Statement Students interested in science, technology, engineering, and mathematics (STEM) typically take introductory courses such as calculus or general chemistry during their first term as undergraduates. Such courses are often perceived as “weeding-out” students, and indeed, previous research has established an association between receiving low grades in these courses and a decreased probability of obtaining a STEM degree. We provide evidence that these courses may disproportionately drive underrepresented minority students out of STEM, even after controlling for academic preparation in high school and intent to study STEM. Thus, introductory STEM courses are institutional structures that may exacerbate disparities in STEM education and, as such, equity issues must be central in efforts to redesign and rebuild them.

## Introduction

Science, technology, engineering, and mathematics (STEM) requires equity, diversity, and inclusion. When these are lacking, public health is hurt (1), scientific innovation and creativity are reduced (2, 3), and economic growth is hampered (4). Moreover, some feel an ethical imperative for STEM pathways to be accessible to all identity groups. Unfortunately, we are faced with the stubborn persistence of STEM disparities (5) that urgently require solutions.

Disparities in STEM education are well-documented. In 2018, women earned 58% of all bachelor’s degrees, but only 36% of STEM bachelor’s degrees (6). In 2017, Black, Hispanic, and Indigenous individuals comprised 30% of the US population, 34% of STEM-intending incoming college students, and yet merely 18% of undergraduate STEM degree recipients (7). These alarming figures suggest that colleges and universities are exacerbating disparities, consistent with the fact that Black and Hispanic students are more likely than their white peers to switch out of a STEM major (8).

We approach our present study through the lens of institutional transformation. A first step in transforming an institution through an equity lens is to identify structures that may inhibit diversity. A next step is to quantitatively analyze whether those structures in fact disproportionately impact marginalized students. In the context of STEM education, introductory STEM courses perceived as “gatekeeper” or “weed-out” courses can be flagged as a potential source of inequity. Student performance in such courses is associated with STEM degree attainment (9). However, there is a reason to believe this association is not neutral with respect to gender and race. That is to say, introductory STEM courses might have a greater negative impact on gender and racial/ethnic minorities (10–13). Our study contributes to the aforementioned next step by providing a quantitative analysis of introductory courses and STEM pathways.

### Research questions

Our work asks: within the framework of a large, multi-institutional database, to what extent do a student’s race/ethnicity, their sex, and their number of Ds, Fs, and/or course withdrawals (hereafter, DFWs) for first term STEM classes impact STEM graduation? Two key aspects of our research question are that (1) it addresses intersecting identity categories and (2) it investigates disparate impact of systems based on those categories. As a secondary research goal, we explore the impact of using a three-level versus a five-level coding scheme for race/ethnicity, which addresses the importance of the ways that race is discussed and categorized in education.

As noted above, associations between STEM degree completion and sex, race/ethnicity, and academic performance have been established, but ours is the first study to draw out interactions that indicates a disproportionate negative effect of introductory courses on minoritized students. To facilitate interpretation of our logistic model, we also will calculate predicted probabilities of STEM degree attainment for students from various demographic categories. The results are a sobering reminder of the progress that must be made with equity, diversity, and inclusion in STEM.

## Methodology

Quantitative studies involving race/ethnicity within a single institution are often challenging because data on minoritized groups is, by definition, limited. For example, suppose a university has a small numbers of records about degree attainment of Black, Native American, and Latinx women. As a result, statistical models involving those groups tend to be unstable. When we consider not just racial/ethnic identity, but its intersection with gender, modeling becomes nearly impossible. The already small counts for racial/ethnic groups are divided into even smaller counts. To navigate this challenge, we draw from a multi-institutional sample that is large enough to enable rich, meaningful statistical modeling, and yet is reasonably homogeneous in the sense that the institutions we select are large, public, research-intensive universities. Specifically, we draw from the Multiple Institution Database for Investigating Engineering Longitudinal Development (MIDFIELD) (14), restricting attention to the six large, public, research-intensive institutions who have data in our selected time frame. The large sample we study is, as we later describe, an order of magnitude larger than those used in previous studies. We are interested in the six institutions for several reasons. First, they serve large numbers of students. Second, they may be more likely to have resources for the institutional changes necessary to mitigate disparities. Finally, research institutions have power and positionality within higher education that may enable them to disseminate models of success to other institutions.

### Modeling

We use a multiple logistic regression framework to model students’ attainment of a STEM degree. Our usage of MIDFIELD data allows us to capitalize on larger sample sizes than if we were to work with a single school’s data. However, this large sample size (*N* = 109,070) comes with several hazards, including a risk of overfitting and the possibility of artificially small *P*-values and narrow confidence intervals. To address the first hazard, we use a 80% training/20% testing split of the data set, stratified along sex, race, and institution (15). To address the second hazard, we use techniques for combating the multiple comparison/simultaneous inference problem (16).

We proceed with model construction as follows. We require each model to contain three specific covariates (high school GPA, ACT composite score, and STEM degree intent, all discussed in more detail below) as well as university attended, which we treat as a nuisance variable. We use step-wise fitting of the testing data to drive the inclusion of other terms from a candidate pool (also described further below), using Akaike information criterion (AIC) for selection.

In addition to presenting estimates for model coefficients, and in order to make the models more easily interpretable, we report estimates of STEM degree attainment probability for STEM-intending students. More specifically, we assume the average values of high school GPA and ACT composite for STEM intending students (3.57 and 26, respectively) and we calculate STEM degree attainment probability as a function of race, sex, and number of STEM DFWs received. Finally, we take a weighted average of the probabilities across the six institutions in order to arrive at a final estimate. All statistical analysis was completed using R, version 4.2.0 (17).

#### Statistical significance

For each coefficient we calculate, we report the SE and the unadjusted *P*-value and, in some cases, 95% CIs. To guard against Type I errors (false discoveries) associated both with the number of tests and large sample sizes, we construct separate testing families for each model’s coefficients. We make use of the False Coverage-Statement Rate method for selective interval construction (18) at 5%. This method strikes a balance between frequentist and Bayesian approaches while directly dealing with the selection of important terms in fitting a regression model. For the probability profiles, we use a more conservative approach for CI construction, namely, Šidák’s method, to control the family-wise Type I error rate at 5%.

### Data context

As mentioned, we make use of MIDFIELD (14). Comprising 20 colleges and universities with engineering programs from across the United States, the database holds *all* student records (not just engineering students) who attended each school as reported by its registrar. The records span 30 y, from 1988 to 2018. While there are more than 1.7 million student records in MIDFIELD, we restrict our attention to students who began college between 2005 and 2012, inclusive. The 2012 limit guarantees that we can study a 6-y time horizon after each student began college. The starting point of 2005 provides more consistency in ACT and SAT score data, since we avoid years in which there were significant changes in how these scores are calculated. Finally, this time frame situates our data after (9), while overlapping with the Mathematical Association of America’s national calculus student study (10) and many of the studies reported in ref. (12).

During our time frame, there are 12 participating schools who provided student information. However, we remove two schools whose student demographics contained in MIDFIELD differ substantially from their publicly reported ones. We remove a third school for which there were only 27 students who had complete records for the terms we use in our models (the other schools had counts in the thousands). Finally, to make a more homogeneous sample, we removed another three schools which are not R1/R2 universities in the Carnegie classification system, leaving us with six schools of similar type: large, public, and research-intensive.

### Model terms

Our final data set consists of *N* = 109,070 students with complete information on our model variables. We use stratified sampling along university attended, sex, and race to form the training set (*N* = 87,231) and testing set (*N* = 21,839).

#### STEM degrees

Our dichotomous model response variable is the attainment of at least one undergraduate STEM degree. For this, we must specify which fields are in STEM. The Congressional Research Service states that “the lack of a common definition for STEM has contributed to confusion, and even contradictory findings” (19). One commonly used definition of STEM comes from the National Science Foundation (NSF) and includes physical sciences, life sciences, mathematical and computational sciences, engineering, as well as certain social science fields (such as political science, economics, and psychology). As our central research question involves the role of first term physical, life, and mathematical courses required of most science and engineering majors, we use a narrower definition of STEM. In particular, we do not include social science fields. MIDFIELD contains Classification of Instructional Program (CIP6) codes for all students’ majors (intended and declared) as well as degrees earned. Based upon the CIP6 code, we coded students dichotomously as earning either at least one or zero undergraduate degrees in a STEM field. In our sample, we find the STEM degree attainment rate to be 15.5% (*N* = 16,958) across all students.

#### Covariates

Our first covariate deals with students’ intent on obtaining a STEM degree. We used the aforementioned CIP6 classification scheme for the program each student was reported as being in during their first term. Given our interest in STEM-intending students, we used a reverse coding scheme. The reference class represents students who intended to get a STEM degree. Approximately 68% (*N* = 74,303) did not intend to pursue a STEM degree at matriculation while almost 32% (*N* = 34,767) did. Nearly 6% of the total students (*N* = 6,455) switched into STEM programs and a little over 15% of students (*N* = 16,993) switched out of STEM programs.

We control for past academic preparation via each student’s high school GPA (20) and ACT composite test score (standardizing both before use in models). GPA varies from 1.0 to 5.0, with sample arithmetic mean of 3.42 (SD of 0.56) and sample median of 3.49 (median absolute deviation 0.52). In the event that a student did not have an ACT composite score, we generate one using that student’s SAT scores and a concordance table (21). Students missing both ACT and SAT scores were removed from the sample at an early stage. ACT composite score varies from 3 to 36, with sample arithmetic mean of 24.52 (SD of 4.02) and sample median of 25 (median absolute deviation of 4.45). This makes the reference class have a high school GPA of 3.42 and an ACT composite of 24.52.

#### University attended

As previously discussed, we use a fairly homogeneous group of six universities that are all public, 4-y schools classified as either R1 or R2 by the Carnegie classification system (none are minority serving institutions). Incorporating individual university effects in our models creates a tension. One appeal of using a large database such as MIDFIELD is to allow for analysis of intersecting identity categories that may not be possible at a single university due to small sample sizes. As we discussed earlier, there may only be a handful of students in the data having certain combinations of sex and race/ethnicity. Fragmenting the data further by incorporating a university effect works against the appeal of a large data set, especially when using a training/testing split. The small cell sizes can lead to an increase in the size of SEs for some terms.

To strike a balance between inclusion of university effects and over-fragmentation of data, we treat the university attended as a nuisance attribute. In particular, we treat the six universities as having fixed effects and as not central to our core research questions. To state in an equivalent way, we are using university attended as a blocking attribute in an ANOVA. As such, we will not construct CIs for these effects.

To choose the reference level for the university-attended variable, we look at university level measures for the six schools, namely, the means of high school GPA, ACT composite, and DFW count, the proportion of students intended to get a STEM degree, and the proportion of students graduating with at least one STEM degree. We also include the proportions of sexes and races. Using this data, we look at both one- and two-cluster classifications along these dimensions. University D is the closest to the centroid for a two-cluster solution and, by just a slim margin, the second closest in a one-cluster solution. Thus, we use University D as the reference class for this variable.

#### DFW count

In many studies of higher education, the terminology of “introductory STEM courses,” is used to encapsulate a small set of key courses such as Calculus I. However, those key courses are not necessarily students’ first contact on campus with STEM disciplines. In our own study, when we speak of introductory STEM courses, we have in mind a broader definition, namely, the STEM courses that a student first took at their university. While MIDFIELD contains each student’s grades for all courses, we restrict attention to course outcomes in any core STEM fields (mathematics/statistics, chemistry, biology, physics, computer science, and related technology fields) during each student’s first term (semester or quarter). We then tabulate the number of DFWs received by each student. We restrict attention to students who took at least one STEM course during their first term. Among these, approximately 72% of students have no DFWs, about 20% have one, and remaining 8% have two or more; see Table 1. Hereafter, we refer to this metric as the DFW count.

**Table 1.** Core STEM DFWs in first term data from MIDFIELD (14).

Zero DFWs	77,996 (71.51%)
One DFW	21,762 (19.95%)
Two DFWs	7,018 (6.43%)
Three or more DFWs	2,294 (2.10%)

#### Demographics

MIDFIELD reports sex and racial/ethnic identity for each student. Table 2 breaks down our sample by these characteristics for each university. Within each cell, the upper values reflect the number of females while the lower values are those for males. The subtotal column gives the marginal breakdown for sex at each university. The reference class for sex is male in our models.

**Table 2.** University-level breakdown of race/ethnicity, stratified by sex of *N* = 109,070 university students who matriculated from 2005 to 2012. Data come from MIDFIELD (14).

**Female**	**White**	**Asian**	**Black**	**Hispanic/Latinx**	**Native American**	**Subtotal**	**Total**
**Male**							
University A	13,217 (12.12%)	704 (0.65%)	675 (0.62%)	555 (0.51%)	74 (0.07%)	15,225 (13.96%)	36,043
	17,925 (16.43%)	1,351 (1.24%)	691 (0.63%)	761 (0.70%)	90 (0.08%)	20,818 (19.09%)	(33.05%)
University B	7,712 (7.07%)	160 (0.15%)	171 (0.16%)	757 (0.69%)	69 (0.06%)	8,869 (8.13%)	16,025
	6,266 (5.74%)	160 (0.15%)	119 (0.11%)	551 (0.51%)	60 (0.06%)	7,156 (6.56%)	(14.69%)
University C	3,292 (3.02%)	232 (0.21%)	346 (0.32%)	175 (0.16%)	30 (0.03%)	4,075 (3.74%)	6,775
	2,271 (2.08%)	151 (0.14%)	169 (0.15%)	101 (0.09%)	8 (0.01%)	2,700 (2.48%)	(6.21%)
University D	7,459 (6.84%)	588 (0.54%)	151 (0.14%)	915 (0.84%)	75 (0.07%)	9,188 (8.42%)	19,860
	10,438 (9.57%)	614 (0.56%)	182 (0.17%)	1,012 (0.93%)	71 (0.07%)	10,672 (9.78%)	(18.21%)
University E	7,739 (7.10%)	563 (0.52%)	2,009 (1.84%)	666 (0.61%)	60 (0.06%)	11,037 (10.12%)	21,709
	8,242 (7.56%)	614 (0.56%)	1,153 (1.06%)	597 (0.55%)	66 (0.06%)	10,672 (9.78%)	(19.90%)
University F	2,449 (2.26%)	205 (0.19%)	261 (0.24%)	419 (0.38%)	172 (0.16%)	3,506 (3.21%)	6,868
	2,487 (2.28%)	223 (0.20%)	151 (0.14%)	354 (0.32%)	147 (0.13%)	3,362 (3.08%)	(6.30%)
Subtotal	41,868 (38.39%)	2,452 (2.25%)	3,613 (3.31%)	3,487 (3.20%)	480 (0.44%)	51,900 (47.58%)	
	47,629 (43.67%)	3,258 (2.99%)	2,465 (2.26%)	3,376 (3.10%)	442 (0.41%)	57,170 (52.52%)	
Total	89,497 (82.05%)	5,710 (5.24%)	6,078 (5.57%)	6,863 (6.29%)	922 (0.85%)		109,070 (100%)

Given the racial demographics in US higher education as a whole, it is unsurprising that the sample is primarily white. As we have mentioned, we use two classifications of race/ethnicity; the five original levels shown here and a three-level recoding of race/ethnicity as white, Asian, or underrepresented minority in STEM (URM). The URM group includes Black, Hispanic/Latinx, and Native American students. This recoding is consistent with the NSF’s terminology (22), but it is important to acknowledge that Asian students’ representation in STEM does not negate marginalization and oppression, including harmful stereotypes such as the model minority myth. The reference class for race/ethnicity is white.

## Results

First, we present estimates from the two logistic regression models, and second, we report probabilities of STEM degree attainment. As a reminder, the reference class for all of our models is a white, male student, who attended University D intending to get a STEM degree, who has average high school GPA and ACT composite score, and who received no DFWs in their first undergraduate academic term.

### Model I: three-level race/ethnicity

In our first model, we use the a three-level coding of race/ethnicity. We opted to use the three-level coding of race/ethnicity given the aforementioned concerns about small number of observations (see Table 2). The model contains our covariates (GPA, ACT, STEM degree intent), university attended, and main effects for DFW count, sex, and three-level race. There are also two-way interactions for sex and race, sex and DFW count, and race and DFW count. The three-way interaction of race, sex, and DWF count was excluded through the step-wise regression process.

For these model terms, we do not observe any significant multicollinearity, with the largest squared generalized variance inflation factor (GVIF) being approximately 2.08 for the three-level race/ethnicity term. There are some potential outliers in the testing data set, which is to be expected with a data set of this size (*N* = 87,231). We opt to accept them as legitimate.

**Fig. 1.**
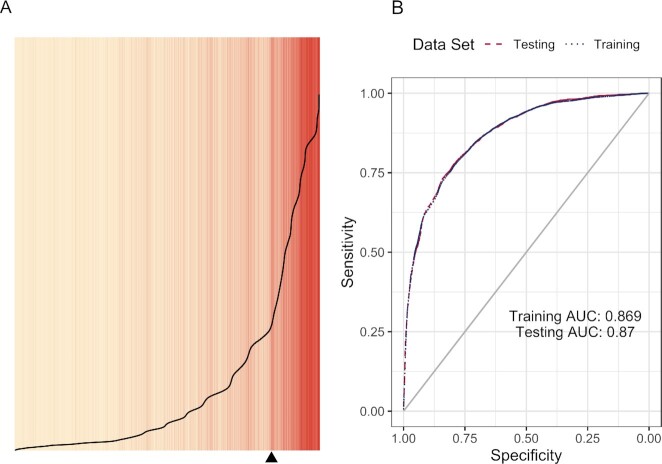
Separation plot (23) (A) for assessment of fit for the first logistic model; ROC curves and AUC values (24) (B).

To assess the fit of this model, we use two approaches: separation plots (23) and Receiver Operating Characteristic (ROC) curves (24) (see Fig. 1). In the separation plot, observations in the training data set are placed from left to right in order of the probabilities predicted from the model. Cases of STEM degree attainment are colored red and cases without STEM degree attainment are colored tan. The vertical axis represents probability, ranging from zero to one. The black curve visualizes the actual probabilities predicted by the model. Finally, the black triangle along the bottom indicates where separation would occur given a perfect model. The model does a fairly good job at sorting the training set with a majority of cases with STEM degrees occurring at or to the right of the triangle. The ROC plot highlights the performance of the model given both the training data and the testing data. The grey diagonal line represents the ROC curve for a model of fair coin flip. Not only do we have consistent model performance between the training and testing data sets (virtually indistinguishable), the model does a much better job than a coin flip. The Area-Under-the-Curve (AUC) values are 0.87 (to two decimal places) for both the training and testing sets. This places our model in the category of “convincing evidence” of classification accuracy (24).

From Table 3, we can see the odds ratios (exponentiated coefficients) and the intercept (odds of the reference class) for all model terms, as well as their *P*-values and 95% adjusted CIs. Of these terms, we only view the coefficients for main effect for Asian and the interaction term of Asian and DFW count to be statistically indistinguishable from unity. The False-Coverage Rate adjusted CIs are suggestive of a slightly reduced model (omitting the interaction of sex and DFW count and the interaction of sex and race) and give a sense of measurement error for the selected coefficients. For the case of no DFW’s, and holding all other aspects constant except for gender and race/ethnicity, a white female, an Asian female, and a URM female have probabilities of STEM degree attainment that are only 0.658, 0.803, and 0.492 times as large as for a white male. Restated, it is 1.52, 1.25, and 2.03 times less probable (respectively) for these female students to get a STEM degree as compared to a white male student, which is consistent with ref. (10). For male and female URM students, respectively, the probability of obtaining a STEM degree is 0.636 and 0.748 times that of a white counterpart (again, holding all other aspects constant). For the interaction of sex and DFW count, the odds ratio of 1.084 suggests that the separate effects of being female and having nonzero DFWs result in an over-penalty, which this interaction term corrects for. However, for the interaction of URM and DFWs, the estimate of 0.870 suggests that there is a differential impact of DFWs for these students.

**Table 3.** Coefficients for logistic regression model of STEM degree attainment using the three-level student race/ethnicity variable.

**Term**	**Estimate**	***P*-value**	**95% Adj**.
	**(SE)**		**CI**
Intercept	3.172 (0.031)	<0.001	(2.998, 3.355)
HS GPA	1.078 (0.015)	<0.001	(1.048, 1.109)
ACT	0.917 (0.012)	<0.001	(0.896, 0.938)
No STEM intent	0.052 (0.027)	<0.001	(0.050, 0.055)
DFW count	0.472 (0.028)	<0.001	(0.448, 0.496)
Female	0.658 (0.027)	<0.001	(0.626, 0.691)
Asian	1.026 (0.065)	0.692	
URM	0.636 (0.057)	<0.001	(0.574, 0.706)
Female and Asian	1.190 (0.095)	0.068	
Female and URM	1.177 (0.076)	0.032	
Female and DFWs	1.084 (0.043)	0.063	
Asian and DFWs	1.030 (0.079)	0.709	
URM and DFWs	0.870 (0.065)	0.033	(0.773, 0.979)
University A	0.122 (0.033)	<0.001	
University B	0.476 (0.036)	<0.001	
University C	1.283 (0.046)	<0.001	
University E	0.193 (0.042)	<0.001	
University F	0.467 (0.048)	<0.001	

### Second model: five-level race/ethnicity

Our second model allows us to explore our secondary research question centering on the impact of using a three-level or five-level coding scheme for race/ethnicity. It is identical to the first model except we change how the race/ethnicity term is coded.

In the second model, we still do not have any significant multicollinearity; the five-level race/ethnicity term still has the the largest squared GVIF (approximately 2.22). As with the first model, we see some potential outliers but retain them in our data.

**Fig. 2.**
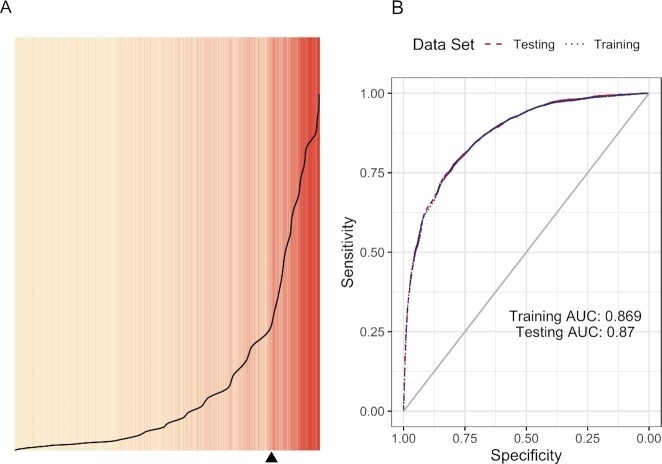
Separation plot (A) for assessment of fit for the second logistic model; ROC curves and AUC values (B).

The difference between the first and second models is essentially indistinguishable for both the separation plots and the ROC curves (see Figs. 1 and 2). Further, the AUC values for the second model are also identical to the first model. In terms of model fit, this suggests that using a three-level coding scheme for race/ethnicity versus a five-level coding scheme does not appear to make much difference.

In Table 4, we have the odds ratios and the intercept term when using the five-level race/ethnicity coding scheme. As expected, all the nonintercept terms not involving race/ethnicity were essentially unchanged in their estimates with the CIs only slightly widening in response to there being more terms. The coefficients for Black, Hispanic/Latinx, and Native American students provide a more nuanced and still pessimistic picture than the single estimate for URM students as a combined category. Furthermore, for these terms, we can start to see the small sample size issue coming into play. The SEs are approximately 1.8, 1.2, and 3.5 times as large as the SE for the URM level in the first model. There is also an increase in SEs for the interaction of race and DFW count. In the first model, this interaction was flagged as significant through our False-Coverage Rate method. In contrast, in our second model, the analogous terms are not flagged. We suspect that given the similarity in the magnitude of the odds ratios between the two models, the difference owes to the small sample sizes in the second model. This difference serves as a reminder that strictly following bright-line rules with regards to *P*-values is an approach that confers challenges and limitations (25).

**Table 4.** Coefficients for logistic regression model of STEM degree attainment using the five-level student race/ethnicity variable.

**Term**	**Estimate**	***P*-value**	**95% Adj**.
	**(SE)**		**CI**
Intercept	3.161 (0.031)	<0.001	(2.972, 3.361)
HS GPA	1.078 (0.015)	<0.001	(1.045, 1.111)
ACT	0.916 (0.012)	<0.001	(0.893, 0.939)
No STEM intent	0.052 (0.027)	<0.001	(0.050, 0.055)
DFW count	0.472 (0.028)	<0.001	(0.446, 0.499)
Female	0.657 (0.027)	<0.001	(0.622, 0.694)
Asian	1.025 (0.065)	0.700	
Black	0.554 (0.104)	<0.001	(0.451, 0.681)
Hispanic/Latinx	0.674 (0.070)	<0.001	(0.587, 0.775)
Native American	0.638 (0.198)	0.023	(0.431, 0.945)
Female and Asian	1.190 (0.095)	0.068	
Female and Black	1.241 (0.129)	0.094	
Female and Hispanic/Latinx	1.204 (0.097)	0.055	
Female and Native American	1.006 (0.262)	0.981	
Female and DFWs	1.085 (0.043)	0.060	
Asian and DFWs	1.030 (0.079)	0.708	
Black and DFWs	0.831 (0.110)	0.092	
Hispanic/Latinx and DFWs	0.907 (0.082)	0.233	
Native American and DFWs	0.838 (0.217)	0.416	
University A	0.123 (0.033)	<0.001	
University B	0.476 (0.036)	<0.001	
University C	1.297 (0.047)	<0.001	
University E	0.195 (0.042)	<0.001	
University F	0.470 (0.049)	<0.001	

### Probability profiles

The interpretation and coordination of multiple coefficients in logistic regression can be a challenging endeavour. To this end, we use the models to produce estimates for the probability that a set of epistemic students will attain a STEM degree. For these students, we have fixed their attributes of high school GPA to 3.57, ACT composite score to 26, and that they intend to get a STEM degree in their first term. The choice of values for GPA and ACT are inline with the average value for these measures in our sample when we restrict to just STEM-intending students. We then vary students along the lines of sex (male or female), race (both three-level and five-level coding schemes), and the number of DFWs (0, 1, or 2). Given that university attended is in the model, we calculate separate tables for each one and present here the averaging of the probabilities, weighted by the proportion of students at each university in the training data set.

Table 5 shows these predicted probabilities using our first model (three-level race/ethnicity). Our findings here are consistent with ref. (9), with the probability of STEM degree attainment for all profiles being less than a fair coin toss (0.5). In looking at the most optimistic case of zero DFWs, we can see that there is difference between white male students and three other groups: URM males, white females, and URM females as shown by the nonoverlapping CIs. Perhaps what is most concerning in Table 5 is what happens when we increase the number of DFWs. Male URM, female URM, and white female students with one DFW have probabilities of getting a STEM degree more similar to those of White males, Asian males, and Asian females with two DFWs. The relative change in probability for white males between zero and one DFW is approximately -31%; for URM males, this value is -41%. For white females and URM females, we see relative changes of -39% and -40%. The relative changes between one and two DFWS for these same epistemic students are approximately -38%, -49%, -36%, and -46%, respectively. These disparities in the relative change in probability suggest that DFWs do not impact students in a uniform way.

**Table 5.** STEM degree attainment probabilities and 95% Šídak corrected CIs for epistemic student profiles using the first model.

	**Three-level**	**Number of STEM DFWs**		
**Sex**	**Race/ethnicity**	**0**	**1**	**2**
Male	White	0.484 (0.464, 0.504)	0.334 (0.313, 0.355)	0.208 (0.184, 0.233)
Male	Asian	0.475 (0.434, 0.517)	0.333 (0.286, 0.380)	0.214 (0.150, 0.278)
Male	URM	0.401 (0.363, 0.439)	0.236 (0.201, 0.272)	0.121 (0.085, 0.158)
Female	White	0.413 (0.392, 0.435)	0.285 (0.261, 0.309)	0.181 (0.151, 0.210)
Female	Asian	0.467 (0.419, 0.516)	0.340 (0.286, 0.395)	0.230 (0.156, 0.304)
Female	URM	0.353 (0.316, 0.390)	0.211 (0.177, 0.244)	0.113 (0.078, 0.148)

When we use our second model that makes use of the five-level categorization of race/ethnicity, we see consistent findings (see Table 6). Note the width of the intervals for Black, Hispanic/Latinx, and Native American students. These intervals tend to be wide due to the small sample sizes, which impact the estimates of the SEs. This is particularly true for Native American students; the lower bounds of the intervals have been truncated to 0 as the calculations actually produce negative values.

**Table 6.** STEM degree attainment probabilities and 95% Šídak corrected CIs for epistemic student profiles using the second model.

	**Five-level**	**Number of STEM DFWs**		
**Sex**	**Race/ethnicity**	**0**	**1**	**2**
Male	White	0.484 (0.463, 0.505)	0.334 (0.312, 0.356)	0.208 (0.182, 0.235)
Male	Asian	0.475 (0.431, 0.519)	0.333 (0.284, 0.382)	0.214 (0.146, 0.281)
Male	Black	0.311 (0.248, 0.374)	0.165 (0.116, 0.214)	0.077 (0.033, 0.122)
Male	Hispanic/Latinx	0.457 (0.408, 0.506)	0.287 (0.236, 0.338)	0.157 (0.097, 0.217)
Male	Native American	0.428 (0.292, 0.565)	0.244 (0.119, 0.369)	0.119 (0*, 0.246)
Female	White	0.413 (0.391, 0.436)	0.285 (0.260, 0.310)	0.181 (0.150, 0.212)
Female	Asian	0.467 (0.416, 0.518)	0.340 (0.283, 0.398)	0.230 (0.153, 0.307)
Female	Black	0.282 (0.230, 0.334)	0.155 (0.111, 0.199)	0.077 (0.034, 0.121)
Female	Hispanic/Latinx	0.421 (0.369, 0.473)	0.269 (0.216, 0.322)	0.153 (0.092, 0.215)
Female	Native American	0.368 (0.235, 0.501)	0.211 (0.094, 0.328)	0.107 (0*, 0.225)

* Confidence interval truncated at zero.

The nuance in Table 6 provides a somewhat more pessimistic view than Table 5. In particular, Black students with zero DFWs have similar probabilities of attaining STEM degrees as white male students with *one or two* DFWs. Black males have relative changes in probability with respect to DFWs of approximately -47% and -53%; Black females have values of -45% and -50%. These discrepancies in the relative changes of probabilities point towards the importance of the interaction of race and DFW count in both models.

## Discussion

For decades, higher education’s efforts to address STEM disparities have focused on “fixing students,” with interventions such as bridge programs, undergraduate research experiences, and remedial/developmental courses (7). These approaches are rooted in perceived deficits in student preparation or interest; they attempt to mold students to better navigate the higher education system as it exists. Despite good intentions, these programs have not reduced attrition among underrepresented minority groups (7). Thus, new approaches are needed, including a critical examination of institutional structures and policies that may inhibit equity. Indeed, major funding agencies such as the NSF, Howard Hughes Medical Institute, and the National Institutes of Health are calling for institutional transformation as part of a multipronged approach to reducing disparities (26–28). We adopted this view in our study through our exploration of the interactions between students’ sex, race/ethnicity, and the impacts of DFWs.

Returning to our first research question, previous work has established the roles that DFWs, race/ethnicity, and sex play in STEM degree attainment (8–10, 12). In contrast, ours is the first study that treats intersecting identity categories. Through our modeling and analysis, we find that DWFs differentially impact students who are women and/or underrepresented minorities. These impacts are negative. Moreover, our work illustrates the severity of these disparities by calculating the probability of degree completion for prototypical students from different demographic groups. In an equitable education system, students with comparable high school preparation, intent to study STEM, and who get Cs or better in all their introductory STEM courses ought to have similar probabilities of attaining a STEM degree. This is not what we observe. White male students have the highest probability of obtaining a STEM degree (48.4%) while female URM students are the least probable STEM graduates at 35.3%. Zooming in more closely, we see that Black female students only have a probability of 28.2% of graduating with a STEM degree. Given the size of our data set and conservative methods of analysis, a gap of this magnitude demonstrates how far we have to go before achieving equity in STEM education. To put our results more plainly, female students and URM students are essentially penalized for attributes over which they have no control. While there is an argument that course grades are at least partially under the control of a student, the roles of teacher and the university should not be ignored. Further, the interaction of students’ race/ethnicity and DFW count points towards the presence of troubling institutional effects. Our study shows that we need to move beyond the “fixing students” mentality. We suggest as a starting point the critical reflection and examination of department, school, college, and university policies and cultures.

In comparing our two models, we find an answer to our secondary research question on the impact of using a three-level versus a five-level categorization of race/ethnicity. Our two models are consistent with each other and suggestive that the choice might be immaterial to answering our primary research question. As with many analysis choices, there are trade-offs. The five-level coding scheme provides more nuance while the three-level coding scheme provides smaller SEs. Our recommendations here are two-fold. First, we encourage other researchers to perform similar comparisons between different coding schemes to see what differences emerge. Such an approach lies with an emerging area known as multiverse analysis (29). Second, researchers should think carefully on what they are attempting to explore and build an understanding of as to whether fine-grain classifications are appropriate. In an ideal world, we would have sufficient numbers to be able to do full analyses of intersecting identities with as fine-grain categorizations as we might wish. However, even using MIDFIELD, sample size issues were still a concern.

### Limitations

There are several limitations which we need to address. First, we have inherited limitations that stem from the fact we are doing secondary data analysis. We are bound by the data reporting choices made by each MIDFIELD participating university registrar as well as the choices made by the MIDFIELD team. While the ideal is to have a consistent coding scheme in any data set, we do not get such a luxury with the MIDFIELD data. The choices of each university create idiosyncratic coding schemes. For example, the race variable contains two categories we omitted from our analysis, Other/Unknown and International, as they are not specific enough for interpretation.

Second, institutions self-select into the MIDFIELD database, and hence constitute neither a random sample nor a nationally representative sample. Indeed, generating a random sample of R1 and R2 institutions and obtaining their student records is, at present, infeasible. On the other hand, the large size of our sample brings benefits that small, random samples do not. For comparison, ref. (9) uses a sample of 7,697 bachelor’s degree intending students, ref. (10) uses 2,266 students, and ref. (8) uses a high of 4,828 students for some of their models. In contrast, our 80% training set (*N* = 87,231) is an order of magnitude larger than all these important studies. Further, the size of our sample in conjunction with the train/test split, and our adjustments for multiple comparisons help to guard against Type I errors, an issue not mentioned in the previous studies.

We hoped that the large size of the MIDFIELD data set would allow for intersectional explorations, especially between race/ethnicity and sex. However, there are still sample size issues when we parse the data by university attended. For example, as shown in Table 2, University C only has eight male, Native American students. Of these, five intended to get a STEM degree and only three actually obtained a one. This small sample size is several orders of magnitude smaller than other racial/ethnic groups and an order of magnitude smaller than other observed counts of Native American students. As we have previously mentioned, the incorporation of university attended essentially weakens the appeal and power of using large multi-institutional databases. As we have shown, these small sample sizes lead to increased standard errors for estimates.

Finally, our response variable—attainment of a STEM degree from a certain university—differs from that of refs. (8, 9). Those studies used self-reports of whether a student attained a STEM degree at *any* university, not necessarily the one at which they originally matriculated. However, from a single university’s perspective, as opposed to a national STEM-pathways perspective, the most salient question is how many students who enter the university actually attain degrees there. The absence of self-reported degree-attainment data in MIDFIELD is, in our view, not a limitation of the present study.

### Future work

More research is needed to establish a causal link and explore interventions that may level the playing field. These future steps are challenging because the classroom experience involves numerous factors: pedagogy, e.g. traditional lecture versus active learning methods (30); class size; assessment, e.g. high-stakes final exams versus mastery grading; course prerequisites and placement; curriculum; and instructor variables such as whether one holds a fixed versus growth mindset (31). In such a complex system, the power of any one explanatory variable to describe student outcomes is likely to be small, so large sample sizes and carefully controlled designs may be needed to observe effects. Regardless, we urge administrators and STEM educators to center equity in all reform efforts, lest they unintentionally improve outcomes for the most privileged students at the expense of those already marginalized.

Our study leaves open several routes for future work. Perhaps most critically, our study, though large in scale, was restricted to public R1 and R2 universities. A large scale national-level study similar to ours could be valuable, but the data is lacking. A large, nationally representative sample is needed. Additionally, it could be valuable to know how outcomes differ by specific intended STEM major, which could also be addressed with nationally representative data. Moreover, analytical frameworks other than ours could be important. For instance, survival analysis of the data could help elucidate the role of time. Finally, we comment that the MIDFIELD database, and indeed many educational data sets, report a binary male/female sex variable. We hope that future data gathering efforts will use self-identified gender, with a richer and more nuanced set of categories.

Supporting projects such as MIDFIELD (14) becomes critical. One way we encourage readers to support is to speak with their own university administration about contributing data to such projects. By building out such databases in careful ways, we hope the above limitations may be resolved for future research.

Though not the focus of our study, our results raise questions about how to go about accounting for prior academic preparedness. In our work we made use of high school GPA and a standardized test score (ACT composite). Recent work (20) has found that high school GPA appears to be a more powerful predictor of college graduation. We found a slight positive association between high school GPA (odds ratio of 1.078 > 1) and graduating with a STEM degree while a slight negative association between ACT composite and the response (odds ratio of 0.917 < 1). We currently have no explanations for these observations. However, they raise the need for researchers to continue questioning whether the benefits of using standardized tests such as the ACT or SAT outweigh the associated problems of systemic racial bias (32).

## Conclusions

This study has contributed to the understanding of diversity and equity in STEM education. We have used a large database of student records to analyze intersecting identity categories. This approach identifies institutional effects where grades differentially impact students by sex and race/ethnicity.

White male students have the highest probability of graduating with a STEM degree when they start college with that intention at 48.4%; however, URM female students only have a probability of 35.3%. Given the size of our data set and conservative methods of analysis, a gap of this magnitude demonstrates how far we have to go before achieving equity in STEM education. We also caution administrators who might gauge equity at their university by comparing internal data to the values in Tables 5 and 6: we ourselves do not consider degree attainment at those rates to represent success. We encourage institutions to take the results from our study and other studies to continue working towards change at multiple levels—course, department, and institution—in order to make STEM pathways diverse, equitable, and inclusive.

## Supplementary Material

pgac167_Supplemental_Files

## Data Availability

The authors were granted access to the data from MIDFIELD. MIDFIELD retains distributional control of the data and encourage researchers to contact them about getting access. Please see https://midfield.online/contact/.
